# Multispecies transcriptomics reveals influenza A virus modulation of *Streptococcus pneumoniae* EF3030 infection in human lung epithelium and murine lung

**DOI:** 10.1128/msphere.00815-25

**Published:** 2026-01-26

**Authors:** Adonis D'Mello, Erin Y. Earnhardt, Jessica R. Lane, Jennifer L. Tipper, Eriel Martínez, Federico I. Prokopczuk, Hansol Im, Holly N. Roussey, Kevin S. Harrod, Carlos J. Orihuela, Hervé Tettelin

**Affiliations:** 1 Department of Microbiology and Immunology, Institute for Genome Sciences, University of Maryland School of Medicine 12264, Baltimore, Maryland, USA; 2 Department of Anesthesiology and Perioperative Medicine, Division of Molecular and Translational Biomedicine, The University of Alabama at Birmingham School of Medicine 9967, Birmingham, Alabama, USA; 3 Department of Microbiology, The University of Alabama at Birmingham 9968, Birmingham, Alabama, USA; University of Michigan, Ann Arbor, Michigan, USA

**Keywords:** *Streptococcus pneumoniae*, influenza, co-infection, transcriptomics, primary human lung cells

## Abstract

*Streptococcus pneumoniae* (Spn) is typically an asymptomatic colonizer of the nasopharynx, but it also causes pneumonia and disseminated disease affecting various host anatomical sites. To delineate host-pathogen transcriptional interactions during pneumococcal (EF3030) and influenza A (pH1N1) coinfection, we used primary differentiated human bronchial epithelial cells (HBEC—three human donors) in a transwell monolayer model at an air-liquid interface, and a mouse pneumonia model, profiled with multispecies deep RNA-seq and NanoString nCounter as complementary models. Distinct pneumococcal gene expression profiles were observed in the presence and absence of influenza in HBEC infection. Influenza coinfection enabled significantly greater pneumococcal growth and triggered the differential expression of bacterial genes corresponding to multiple metabolic pathways. Notably, a fundamentally altered bacterial metabolic state and a greater nutrient availability were observed when coinfecting with influenza. Downregulation/deletion of sialic acid utilization genes promoted EF3030 proliferation during mono/coinfection with pH1N1 on HBEC. Surprisingly, HBEC transcriptomes were only modestly perturbed by infection with EF3030 alone relative to changes resulting from influenza A infection or coinfection. Influenza-infected HBEC transcriptomes showed significant loss of ciliary function, with changes in host defense, microtubules, and extracellular matrix (ECM). Some of these findings were confirmed in the murine lung infection model. Influenza-mediated changes in the host epithelium transcriptome also contribute to bacterial invasiveness. This included downregulation of genes involved in expressing cilia and increased ECM degradation. Ultimately, we identified novel genes and pathways involved in the dynamics of epithelium-influenza-pneumococcus coinfection, such as EF3030 metabolic regulons (NanR, LacR, etc.) and epithelial protein families (keratins and matrix metalloproteases). We conclude that influenza infection promotes a pneumococcal metabolic shift, allowing for transition from colonization to disseminated disease and an exacerbated breakdown in the epithelium, potentially permitting enhanced EF3030 infection and dissemination.

**IMPORTANCE:**

Transition from pneumococcal colonization to invasive disease is not well understood. Studies have shown that such a transition can occur as a result of influenza A virus (IAV) coinfection. We investigated the pneumococcal (serotype 19F, strain EF3030, and isogenic mutants) and airway epithelial transcriptomes with and without IAV (A/California/07 2009 pH1N1) infection. Pneumococcus and influenza coinfection leads to enhanced bacterial transcriptional programs related to growth, nutrient availability, and energy biosynthesis, suggesting conversion to an invasive phenotype. Influenza-induced secondary EF3030 infection influences human bronchial epithelial cell (HBEC) microtubules and extracellular matrix. Notably, sialic acid (NanR) utilization is a central regulon in EF3030 mono/coinfection with pH1N1 on HBEC. Downregulation of sialic acid utilization during influenza coinfection improved Spn pathogenicity *ex vivo* but did not alter disease *in vivo,* suggesting other metabolic cues are also important. This study uncovers critical metabolic features of the EF3030-pH1N1 interface to inform how Spn proliferates during IAV coinfection.

## INTRODUCTION

*Streptococcus pneumoniae* (Spn), a colonizer of the nasopharynx and the most common cause of community-acquired pneumonia, is also one of the most frequent agents responsible for bacterial coinfection following influenza virus infection (1). Considerable historical evidence, including that collected during past influenza pandemics and epidemics, has established that influenza infection starkly predisposes the host to secondary bacterial infections (2). Bacterial coinfections of the respiratory tract alongside a virus are characterized by their greater severity and higher mortality rates when compared to those associated with bacterial pneumonia alone (3, 4). Multiple studies have investigated the role of influenza and Spn in superinfections. Preceding influenza infection has been shown to increase pneumococcal burdens in both the upper and lower airways (5). Investigations into the factors that drive host-to-host transmission of Spn revealed increased transmission as a result of influenza pre-infection in mice (5), ferrets, and primates. Interestingly, this resulted in reduced influenza transmission (6). Recently, investigators have taken genetic approaches to identify the bacterial genes required for superinfection. For example, using CRISPRi-seq, Liu et al. showed a requirement for pneumococcal capsule and the adenylsuccinate synthetase gene *purA* (7).

Pettigrew et al. demonstrated that preincubation of influenza with Spn prior to host infection caused an increase in virulence and bacteremia (8). With model systems used herein, Earnhardt et al. showed that influenza-driven regulation of human CFTR (cystic fibrosis transmembrane conductance regulator) leads to airway pathophysiology that contributes to increased Spn burden in the mucosal lining (9). Furthermore, virulence factors produced by Spn and host susceptibility to lung epithelial necroptosis both promote bacterial growth and dissemination (10). Seigel et al. showed that influenza-infected mice increased Spn colonization and aspiration, via influenza neuraminidase cleavage of sialic acid residues from mouse mucin receptors (11). Spn neuraminidases, in turn, are also capable of altering influenza shedding and transmission (12), suggesting an important role of environmental sialic acid for the two pathogens (13). Moreover, Hentrich et al. showed that sensing and metabolism of sialic acid are maintained by pneumococcal response regulator CiaR (14). Several major human genes and chemokines have been identified as vital components of the host response. These include IL-17, IL-23, IL-12, CD200, and type I interferon genes (3, 15). Their overexpression is also thought to contribute to the excessive inflammatory response that characterizes secondary bacterial infection and contributes to its severity. So far, the bulk of research efforts have been focused on the bacterial virulence determinants required for infection and superinfection, and the host response to infection by the influenza virus and Spn. A comprehensive study on how Spn alters specific gene expression and its metabolome in the context of influenza infection, exclusively on the isolated primary bronchial epithelium, remains unknown, and this is likely to reveal new insights into the basis of disease.

We hypothesized that influenza infection not only skews host defense, rendering the airway more permissive to pneumococcal persistence, but also alters the host environment in a manner that alters and promotes bacterial virulence. Using multispecies RNA-seq, we tested this hypothesis by exploring the transcriptome of Spn strain EF3030, a modestly virulent strain of Spn, during infection of differentiated primary human lung epithelial cells and infection of mouse lung tissue, in the presence and absence of influenza A virus (IAV) pH1N1. Our results shed light on the major genes and pathways involved in the superinfection process from a bacterial and host perspective.

## RESULTS

### Spn EF3030 gene expression is reshaped by influenza infection in human lung epithelial cells

**Fig 1:**
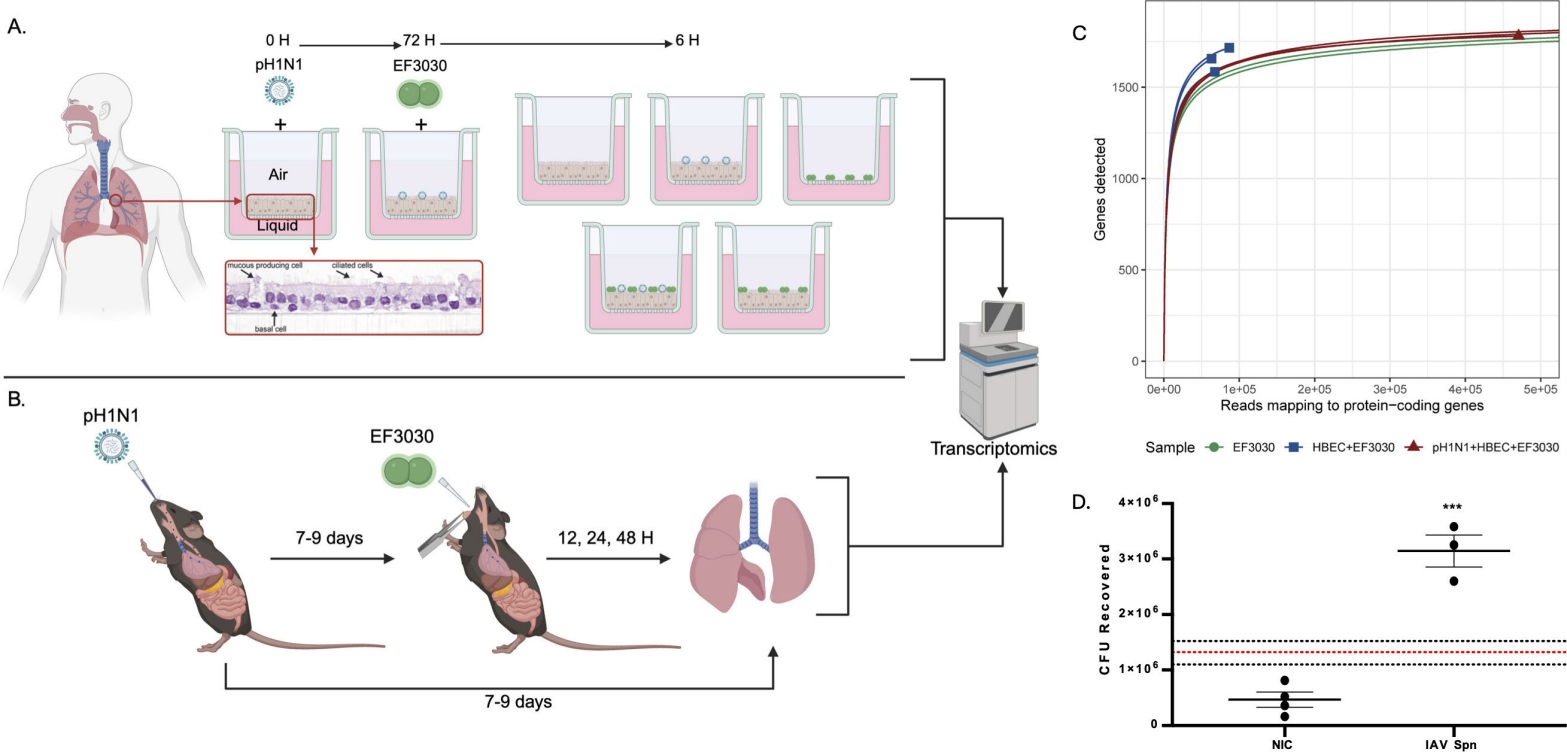
Overview of the study. (**A**) Airway epithelium was isolated from lung transplant material from a single human donor and cryopreserved. HBECs were thawed and cultured in media for 2 days until confluence was reached and transferred to an air-liquid interface (ALI) system, stimulating differentiation into epithelial phenotypes. HBECs were then infected or mock infected with pH1N1 virus for 72 h, followed by Spn EF3030 infection for 6 h. Conditions included one coinfection condition with pH1N1 and EF3030, two monoinfections of either pH1N1 or EF3030, and two controls of uninfected HBECs and EF3030 cells grown alone in the ALI system. RNA was isolated from experimental replicates of these five conditions and sequenced using the Illumina NovaSeq 6000 system, followed by RNA-seq read mapping to the EF3030, pH1N1, and human genomes, with analysis using traditional bulk RNA-seq approaches. (**B**) Nine-week-old mice were intranasally infected with pH1N1. Between 7 and 9 days post-infection, mice were challenged intratracheally with EF3030. Conditions included one coinfection condition with pH1N1 and EF3030 and a monoinfection of pH1N1. Mice were euthanized at 12/24/48 h time points and had their lungs harvested. RNA isolation, sequencing, and analysis were performed similarly to those in HBEC infections. Images were created with BioRender.com. (**C**) Rarefaction curves of EF3030 samples. Samples whose curves plateau indicate sufficient sequencing depth for the majority of EF3030 genes analyzed. (**D**) EF3030 colony-forming unit (CFU) counts for HBEC + EF3030 (no-influenza control, NIC) vs. pH1N1 + HBEC + EF3030 (IAV Spn). Dotted lines represent the mean of EF3030 recovered from 1,000 CFU EF3030 inoculated into BEGM media (no HBECs, red) and its standard error (black). Data were analyzed using an unpaired two-tailed *t*-test. *** indicates *P* ≤ 0.0005.

Primary differentiated human bronchial epithelial cells (HBEC) grown on transwells from three donors were infected with IAV pH1N1 for 72 h followed by superinfection with Spn EF3030 for 6 h (Fig. 1A; see Materials and Methods). Gene expression profiling was performed with multispecies RNA-seq and the NanoString (Bruker) nCounter platform with Spn gene target probes (16). Additionally, we infected C57BL/6 mice with IAV pH1N1 for 7–9 days, followed by superinfection with Spn EF3030 for periods of 12 h, 24 h, and 48 h (Fig. 1B; see Materials and Methods). Infected lungs were harvested and processed for multispecies RNA-seq in a similar manner. One caveat of multispecies RNA-seq that commonly occurs is significantly lower sequencing read amounts for the less dominant species, *Spn* EF3030, in this case. As a result, we were unable to obtain and analyze *Spn* EF3030 transcriptomes from mouse-infected lungs. Henceforth, all proceeding analyses on EF3030 transcriptome data were performed exclusively on the infected HBEC transwell samples in Fig. 1A. Read mapping statistics for all three species and their normalized expression counts (VST) are provided in Tables S1 and S2, respectively.

**Fig 2:**
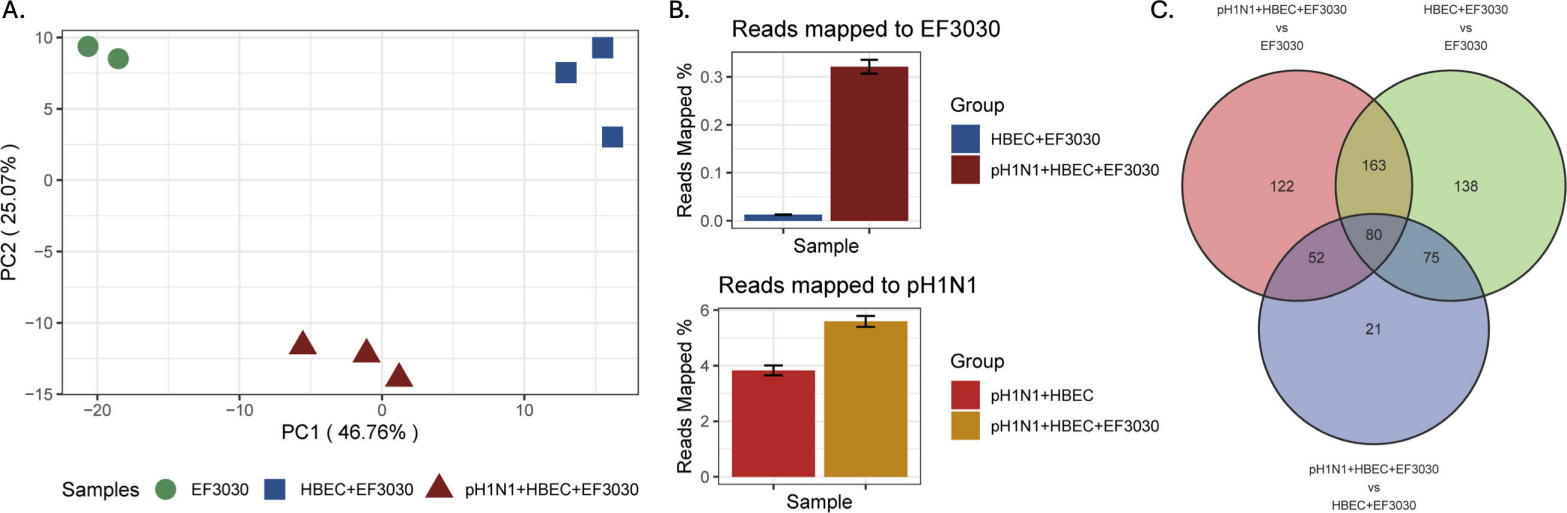
Overview of pneumococcal EF3030 RNA-seq transcriptomes. (**A**) PCA plot of EF3030 gene expression profiles. (**B**) Read mapping percentages to the EF3030 and pH1N1 genomes. (**C**) Venn diagram of genes identified from three differential expression analysis comparisons of EF3030 gene expression.

To ensure adequate RNA sequencing depth for robust interrogation of the EF3030 transcriptome from transwells, we generated saturation curves for all Spn-infected samples (Fig. 1C). We observed high coverage for EF3030 in media only (inoculum) and pH1N1 + HBEC + EF3030 samples, as those saturation curves plateaued for over 95% of EF3030 protein-coding genes. Due to the colonizing (rather than invasive) nature of EF3030, fewer Spn cells were present in HBEC + EF3030 samples, and their saturation curves only just approached a plateau in line with the other samples. Additionally, we observed a 6× increase in bacterial CFUs from an apical wash of infected transwells in our experiments following identical infection protocols (Fig. 1D). Reproducibility of RNA-seq data obtained from biological replicates was evaluated using a principal component analysis (PCA) (Fig. 2A) that showed three tight clusters corresponding to the different EF3030 sample types. PC1 captured ~47% of the overall variation in EF3030 gene expression, with the largest difference observed between EF3030 grown in media alone and EF3030 encountering the human lung epithelium (no influenza: HBEC + EF3030). PC2 also captured ~25% variation separating EF3030 gene expression in pH1N1 coinfections of the human lung epithelium toward the bottom of the PCA. This also suggests that certain EF3030 genes might respond to the virus directly or indirectly during the lung epithelial cell infection process. One of the key observations we made from the reads mapping to the EF3030 genome (Table S1) was that the proportion of reads that mapped to EF3030 was significantly higher for pH1N1 + HBEC + EF3030 relative to HBEC + EF3030 (Fig. 2B), with ~30× more bacterial reads mapped from pH1N1 + HBEC + EF3030 samples. A similar observation was made for pH1N1 reads in pH1N1 + HBEC + EF3030 compared to pH1N1 + HBEC, with ~50% more reads mapping. These results are likely not directly representative of true measures of CFUs of EF3030 in these samples, but are in line with our previous observations (Fig. 1D), as higher EF3030 read proportions imply increased EF3030 CFUs. This also correlates with known increases in pneumococcal burden upon influenza infection (9).

### EF3030 undergoes distinct metabolic shifts during IAV coinfection

To identify the major EF3030 gene responses to transwell epithelia and epithelia with pH1N1, we performed a differential expression analysis using DESeq2 (17). We queried differentially expressed (DE) genes for the comparisons of HBEC + EF3030 relative to EF3030 in media only, pH1N1 + HBEC + EF3030 to EF3030 in media only, and pH1N1 + HBEC + EF3030 to HBEC + EF3030, considering only DE genes that passed a DEseq2 FDR cutoff of ≤0.05 and an absolute log_2_ fold change (LFC) of ≥1 (Fig. 2C).

163 DE genes were shared between the HBEC + EF3030 vs. EF3030, and pH1N1 + HBEC + EF3030 vs. EF3030 comparisons. This suggests these genes are differentially expressed in EF3030 upon encounter with the human lung epithelium. There were 80 DE genes in all three comparisons, suggesting regulation of these EF3030 genes constitutes a core host-pathogen interaction process. 52 DE genes were also shared between the pH1N1 + HBEC + EF3030 vs. EF3030 and pH1N1 + HBEC + EF3030 vs. HBEC + EF3030 comparisons, suggesting these EF3030 genes are regulated specifically due to the presence of pH1N1 in coinfection. The Spn EF3030 DE gene lists and the intersections detailed here are provided in Table S3.

**Fig 3:**
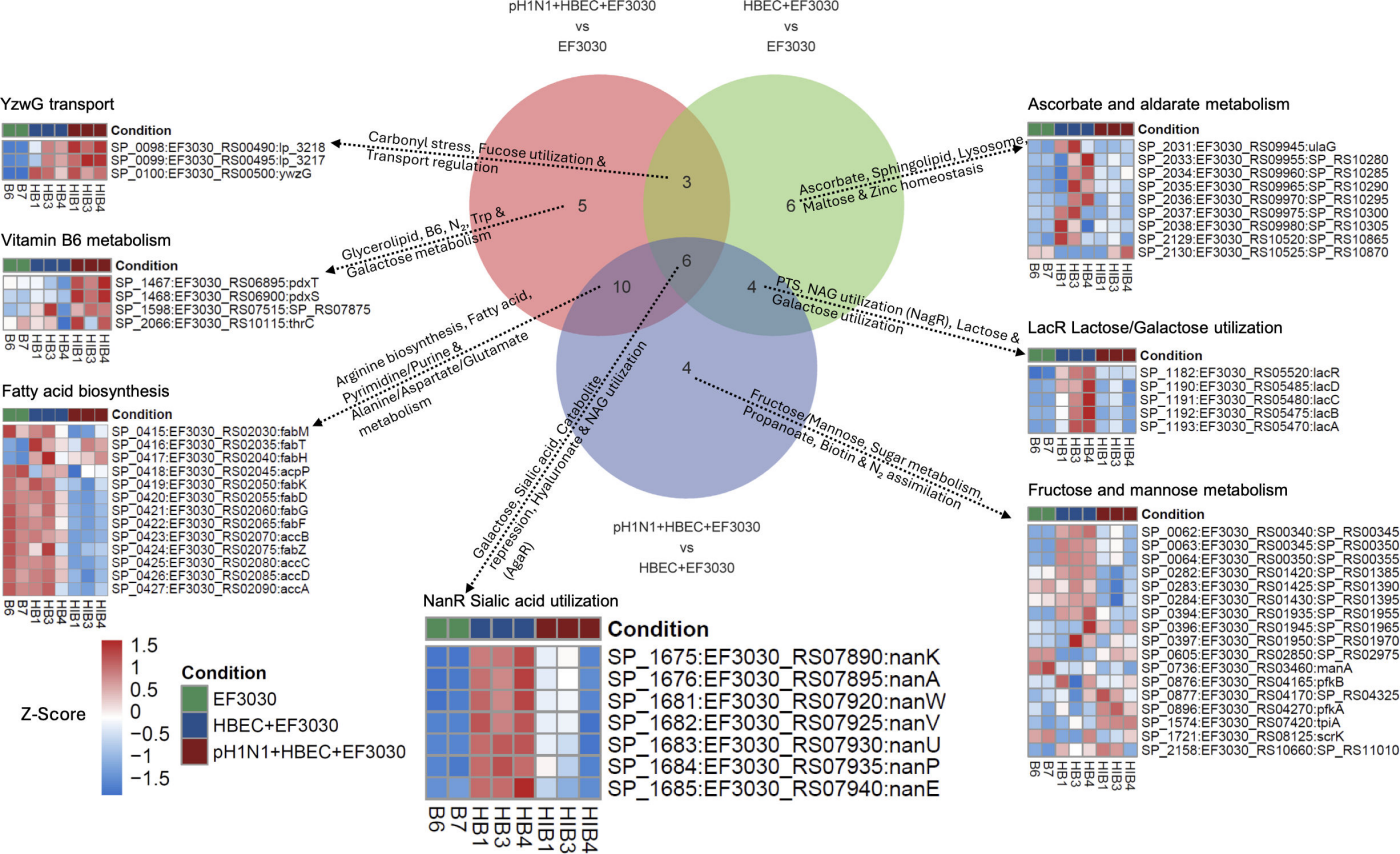
Gene expression profiles of RNA-seq differentially regulated pneumococcal metabolic regulons. Venn diagram of EF3030 biological pathways identified from differential expression analysis comparisons of EF3030 gene expression. Text around arrows briefly describes the affected biological pathways in each set of the Venn diagram. Each arrow points to a single representative pathway from the set, highlighting condition-specific expression patterns, exhibited as a Z-scored heatmap of EF3030 genes expressed in the pathway.

Using the KEGG and RegPrecise databases, differentially regulated pneumococcal pathways were determined for each comparison: 24 pathways for pH1N1 + HBEC + EF3030 vs. HBEC + EF3030; 24 for pH1N1 + HBEC + EF3030 vs. EF3030; and 19 for HBEC + EF3030 vs. EF3030 (Fig. 3). An overview of the regulated pathways for each subset of the Venn diagram is described on the arrows pointing to a representative pathway in the form of a Z-scored heatmap of EF3030 gene expression. A select few pathways were uniquely enriched for each of the comparisons. For HBEC + EF3030 infection relative to in media EF3030, we saw activation of many ascorbate, maltose, sphingolipid, and zinc homeostasis genes. For pH1N1 + HBEC + EF3030 infection vs. EF3030, we noted activation of vitamin B6, nitrogen, and galactose metabolism, and repression of tryptophan metabolism. For secondary EF3030 infection, that is, pH1N1 + HBEC + EF3030 vs. HBEC + EF3030, we observed repression of fructose/mannose, sugar, propanoate, and biotin metabolism, as well as activation of nitrogen assimilation (GlnR regulon). Six pathways were enriched in all comparisons, indicating that certain gene sets play a dynamic role in HBEC infection and influenza-induced secondary pneumococcal infection. Among these pathways was the NanR regulon of sialic acid utilization (Fig. 3), consisting of two operons around the same locus, which was largely activated during both mono/coinfection, albeit to different degrees relative to EF3030 in media. We chose to investigate the impact of this regulon, referred to as NanR, during HBEC infection using a double operon knockout EF3030 mutant, detailed later in the text, and hereafter referred to as ∆*nanR*.

We performed an independent validation of EF3030 RNA-seq data (the limiting species in our multispecies assay) using the Nanostring nCounter platform on the same RNA samples. We designed an nCounter panel of 72 pneumococcal genes (Tables S3, 66 genes representing EF3030) and observed strong correlations between their observed expression in RNA-seq and nCounter with Spearman correlation (R) values ranging from 0.81 to 0.9 (Fig. S1A). We also correlated the average EF3030 expression values from RNA-seq (1 HBEC donor) with the average EF3030 expression values from nCounter data obtained from two independent HBEC donors (Fig. S1B and C). We observed correlation values (0.56–0.59) for EF3030 infections, with higher correlations for pH1N1 + EF3030 coinfections (0.82) in both donors.

### Host-glycoconjugate-associated sialic acid is a vital carbon source for EF3030 during bronchial epithelial cell infection

Sialic acid utilization (NanR regulon; Fig. 3) appeared to be a crucial metabolic process as it was upregulated in both EF3030 monoinfection and IAV coinfection, relative to EF3030 in media only. We investigated the metabolic and virulence impact of this regulon using the ∆*nanR* mutant (Fig. S2A, operons deleted: EF3030_RS07890- EF3030_RS07895; EF3030_RS07920- EF3030_RS07940). HBECs from the two additional human donors (designated AEIC or AHJF) were infected using EF3030 under identical experimental conditions as described in Fig. 1A. These included coinfections with pH1N1 and wild-type (WT) EF3030. Interestingly, we observed the growth kinetics of the ∆*nanR* mutant relative to EF3030 in media and found the mutant had a modest fitness advantage over the WT strain (Fig. S2B), with decreased doubling time, during the logarithmic growth phase relative to its WT when measured under rich media conditions *in vitro* (Fig. S2C). Moreover, pronounced better growth was observed during HBEC mono/coinfection with pH1N1 (Fig. S2D), with titers of the ∆*nanR* mutant being 10-fold greater than EF3030 WT during pH1N1 coinfection. This result aligns with the downregulation of the NanR regulon (Fig. 3) explicitly during pH1N1 coinfection relative to monoinfection, specifically on primary HBEC under these conditions. Thus, the influenza-induced downregulation of NanR, much like the deletion of NanR, may have a role in bacterial proliferation during coinfection of HBEC. However, when CFU counts from similar mono/coinfections were determined in murine lungs, the fitness advantage of *nanR* deficiency was not observed (Fig. S2E). This suggests that the NanR-regulated phenotype is restricted to the human epithelium, that other metabolic cues present *in vivo* are equally important and counter Spn outgrowth, or that the overall increased growth kinetics of NanR deletion is restricted by immune cells or other host defense mechanisms during pneumonia.

**Fig 4:**
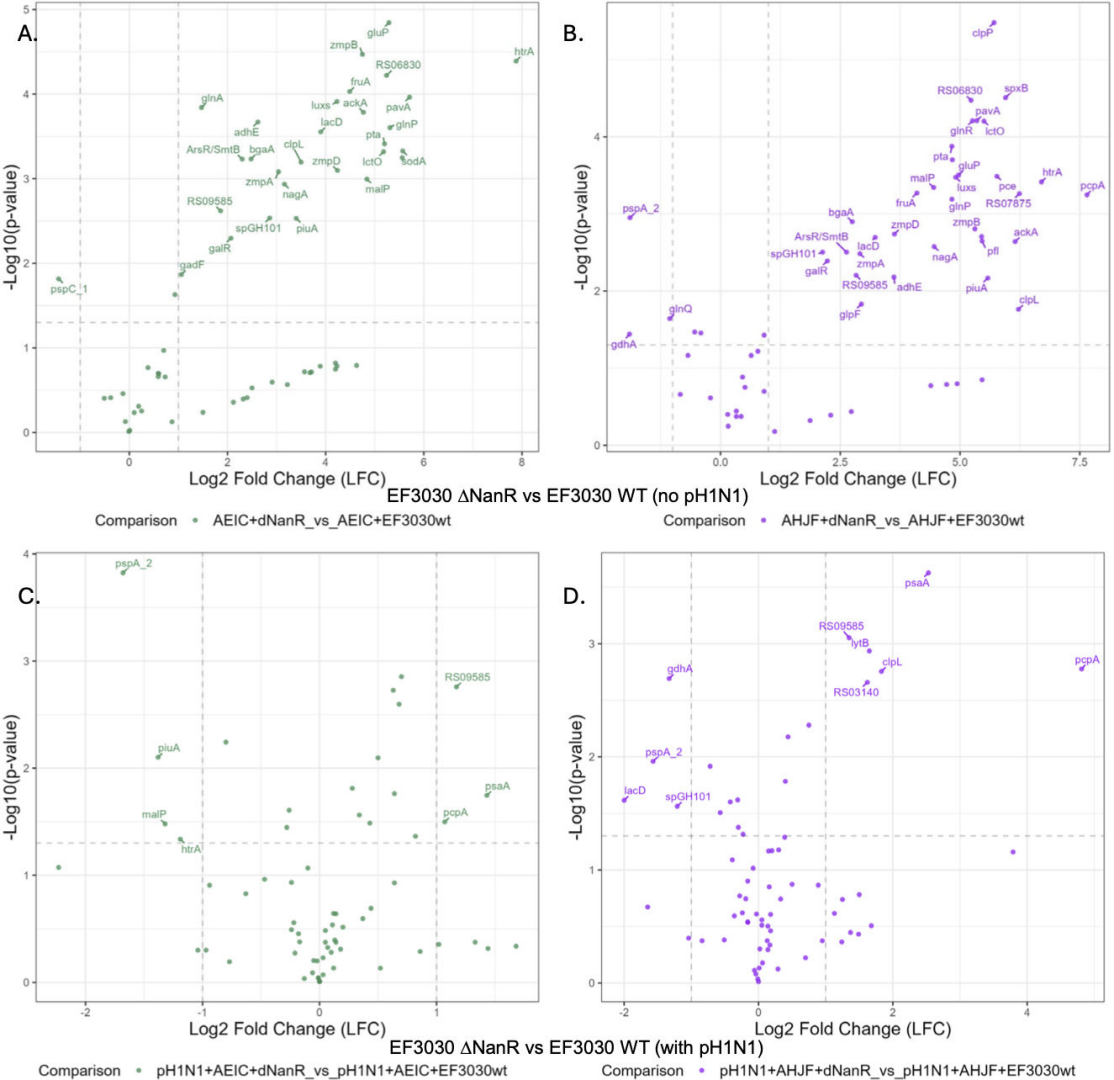
Impact of EF3030 ∆*nanR* mutation on the transcriptomic profile of selected pneumococcal genes during EF3030 monoinfection or pH1N1 coinfection. Volcano plots of the observed expression of selected pneumococcal EF3030 genes from a Nanostring nCounter analysis performed on HBEC samples from two additional human donors (AEIC—left and AHJF—right), each infected with WT EF3030 or double operon knockout mutant EF3030 ∆*nanR*. Samples were infected identically to those described in Fig. 1A. Only significant EF3030 DE genes discussed in the text are labeled with their gene symbols when available, or truncated locus tags. (**A, B**) Volcano plots of EF3030 ∆*nanR* vs EF3030 infection. (**C, D**) Volcano plots of EF3030 ∆*nanR* vs EF3030 infection, with 72 h prior pH1N1 infection.

Using our custom nCounter panel, DE genes were determined for ∆*nanR* vs WT EF3030 monoinfection (Fig. 4A and B) and ∆*nanR* vs WT EF3030 during influenza coinfection (Fig. 4C and D), depicted as volcano plots. There was a substantial impact of the *nanR* deletion during EF3030 monoinfection, with ~33% of the probed genes DE, all being upregulated in both human donors. Of the virulence genes, we observed upregulation of all EF3030 zinc metalloproteases, *zmpA*, *zmpB*, and *zmpD*. Also upregulated were *pavA*, *piuA*, serine protease *htrA*, endo-α-d-N-acetylgalactosaminidase SpGH101, and β-galactosidase *bgaA*. Many metabolic genes were involved in specific operons or biological processes, including glutamine metabolism (glutamine and glutamate transporters *glnP*, *gluP*), lactate metabolism (lactate oxidase *lctO*, tagatose 1,6-diphosphate aldolase *lacD*), fructose metabolism (transporter), galactose metabolism repression (operon repressor *galR*), acetate metabolism (phosphotransacetylase *pta*, acetate kinase *ackA*), N-acetylglucosamine metabolism (*nagA*), and maltose metabolism (maltodextrin phosphorylase *malP*). Other upregulated genes were heat shock protein Clp, autoinducer *luxS*, and acylphosphatase. Other notable genes that were affected on an individual donor basis included virulence genes such as *pspA* and *spxB*, as well as other parts of metabolic regulons such as glutamine (*glnR*). We interpret these results as meaning that EF3030 ∆*nanR*, now incapable of consuming its preferred carbon source, resorts to supplementary forms of feeding with enhanced virulence as a consequence.

Notably, fewer genes were impacted by *nanR* deletion during coinfection with pH1N1. Three genes were upregulated in both donors (choline binding protein *pcpA*, acylphosphatase, and manganese transporter/pneumococcal surface adhesin A *psaA*), and one gene was downregulated (*pspA*). Some metabolic impact was observed on an individual donor basis, albeit not to the extent seen during EF3030 monoinfection. This minor metabolic impact on EF3030 ∆*nanR* during pH1N1 coinfection is most likely indicative of an influenza-induced nutrient-replete environment allowing enhanced EF3030 proliferation and infection. A comprehensive list of the pneumococcal genes probed with the NanoString nCounter platform and their relative expression values in the tested comparisons discussed here is presented in Table S4.

### IAV infection overwhelmingly alters the human lung epithelium transcriptome

**Fig 5:**
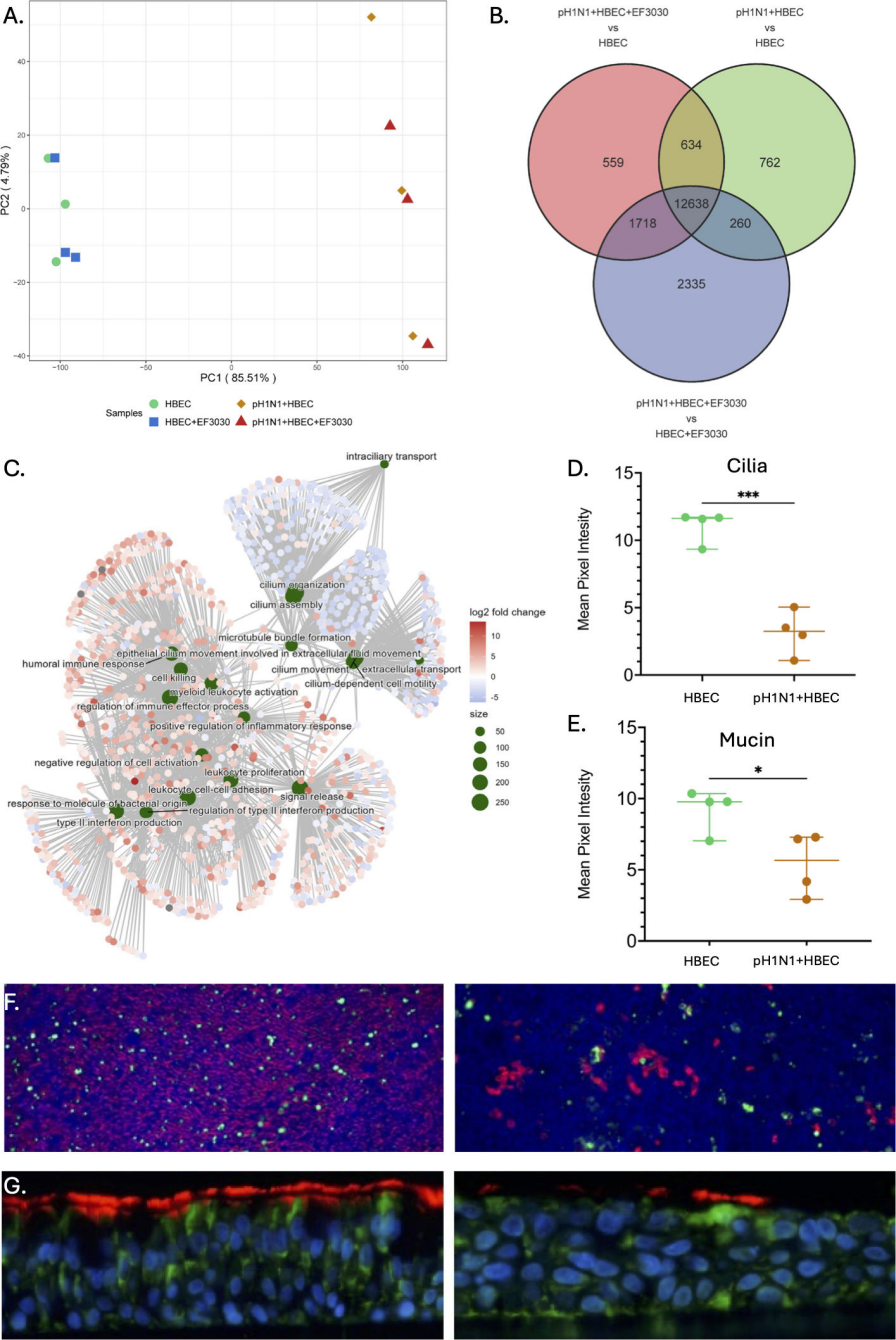
Overview of human HBEC RNA-seq transcriptomes. (**A**) PCA plot of HBEC gene expression profiles. (**B**) Venn diagram of genes identified from three differential expression analysis comparisons of HBEC gene expression. (**C**) Top 10 enriched Gene Ontology (GO) biological processes visualized as a gene-concept network plot: inner green circles represent biological processes and outer circles represent genes colored by LFC for the pH1N1 + HBEC + EF3030 vs. HBEC + EF3030 comparison. (**D and E**) Mean pixel intensity of cilia and mucus staining was quantified in ImageJ and analyzed by an unpaired two-tailed *t*-test. * and *** indicate *P ≤* 0.05 and *P ≤* 0.0005, respectively. (**F**) Immunofluorescence of ALI cultured HBECs: uninfected cells (left); cells infected with pH1N1 for 72 h (right). Red stain represents cilia and green stain represents mucin (MUC5AC)-producing goblet cells, imaged on the Leica DFC 450 C system with the HC PL FLUOTAR10X/0.30 objective. (**G**) Immunofluorescence images of ALI from vertical slices of embedded transwells, under the same respective conditions as panel F (cilia in red and CFTR in green), imaged on the Leica DFC 450 C system with the HC PLAN APO 20×/0.70 objective.

Host conditions included the following: control uninfected human lung epithelial cells (HBEC), HBEC + EF3030, pH1N1 + HBEC, and pH1N1 + HBEC + EF3030. PCA (Fig. 5A) revealed a stark separation along PC1 (~85% of the variation) between pH1N1 + HBEC and pH1N1 + HBEC + EF3030 on the right, and HBEC and HBEC + EF3030 on the left. PC2 captured only ~5% of variation between biological replicates. HBEC and HBEC + EF3030 samples clustered together with almost no difference, suggesting no major influence on the host from only EF3030 infection (6 h). Similarly, within pH1N1-containing samples, no clear separation was seen between pH1N1 + HBEC and pH1N1 + HBEC + EF3030, suggesting that the presence of EF3030 coinfection had no broad effect on the host during pH1N1 infection, potentially due to an overwhelming response to just pH1N1. Using DESeq2 (17), we identified 12,638 human genes specific to pH1N1 infection, with 2,335, 762, and 559 DE genes unique to the comparisons of pH1N1 + HBEC + EF3030 vs. HBEC + EF3030, pH1N1 + HBEC vs. HBEC, and pH1N1 + HBEC + EF3030 vs. HBEC, respectively (Fig. 5B). Only a few genes (<3) were DE with an FDR cutoff of ≤0.05 and an absolute LFC of ≥1 for the comparisons of HBEC + EF3030 vs. HBEC, and pH1N1 + HBEC + EF3030 vs. pH1N1 + HBEC. The list of all HBEC DE genes and subsets is presented in Table S5.

**Fig 6:**
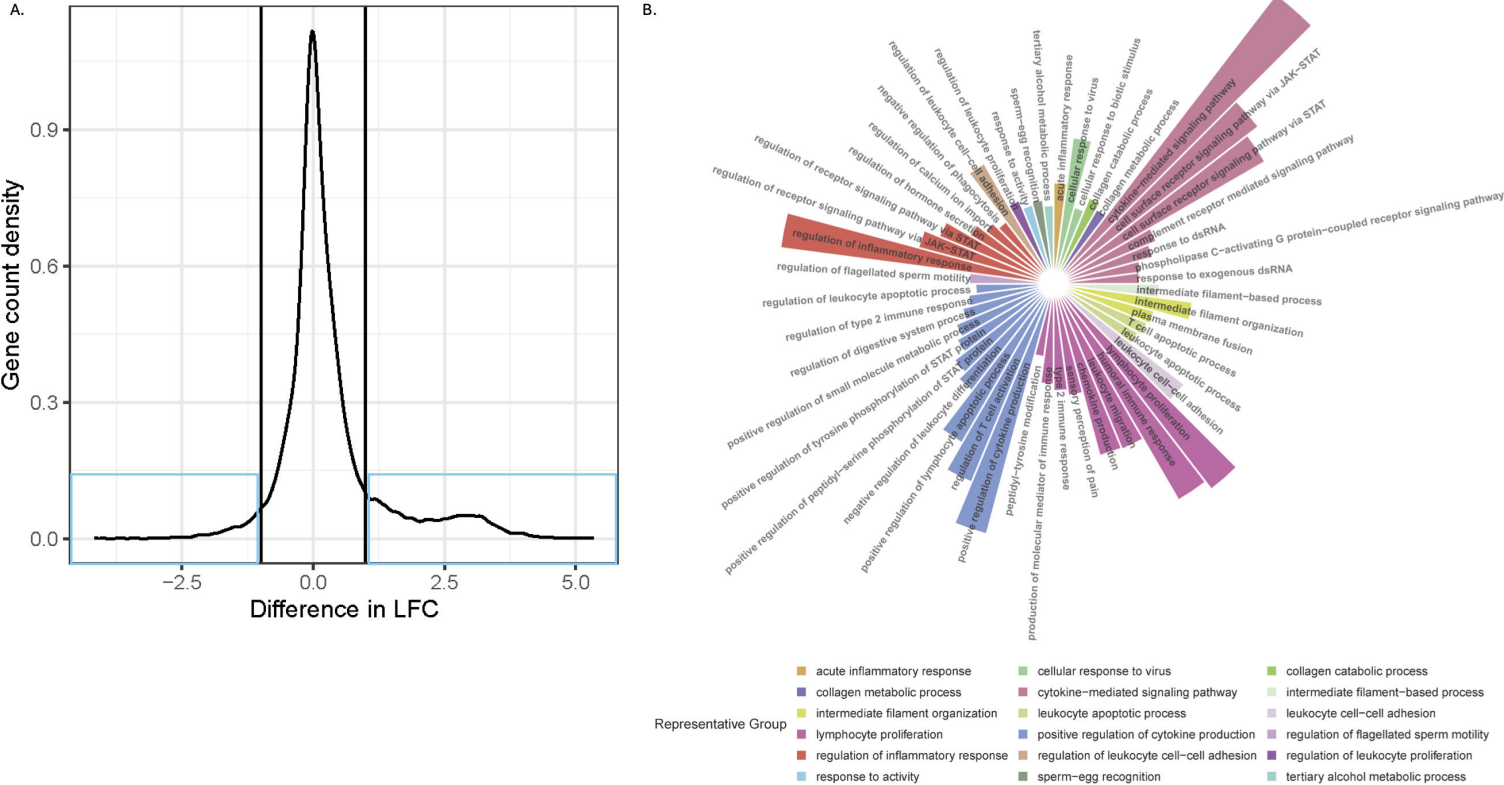
Significantly differentially regulated host genes between HBEC influenza infection and HBEC influenza-induced secondary EF3030 infection. (**A**) RNA-seq LFC differences between HBEC influenza infection (pH1N1 + HBEC vs HBEC) and influenza-induced HBEC secondary EF3030 infection (pH1N1 + HBEC + EF3030 vs HBEC + EF3030). Blue boxes highlight ~2,100 genes with absolute LFC changes ≥1. (**B**) Summarized REVIGO plot of GO biological processes enriched by genes from blue boxes in panel A. Related categories have the same respective color. The size of individual highlights is proportional to GO enrichment-adjusted *P*-values.

As several thousand DE genes were identified during infection/coinfection, typical pathway analyses would be overwhelmed, as they are optimized to work with a typical upper limit of ~3,000 DE genes. Hence, we attempted to identify the broader biological processes involved during both pH1N1 infection and EF3030 + pH1N1 coinfection using GO term analysis using the R package ClusterProfiler v4.0 (18). Associated GO processes for each comparison are listed in Table S5. We saw enrichment of several of the same GO biological processes in all comparisons involving pH1N1 presence vs. pH1N1 absence, mainly involving the upregulation of several cytokine and immune signaling processes, defense responses to bacteria, but also a severe decline in ciliary regulation, ciliary beating, and microtubule pathways (Fig. 5C). Ciliary downregulation was also confirmed and quantified phenotypically by the reduction of cilia and mucin-producing goblet cells using fluorescence microscopy (Fig. 5D through G). To deconvolute the impact of secondary pneumococcal infection (pH1N1 + HBEC + EF3030 vs HBEC + EF3030) relative to influenza infection (pH1N1 + HBEC vs HBEC) among the shared 12,638 DE genes, we compared their LFC differences. We noted that nearly all these DE genes were regulated in the same direction for these two comparisons. However, ~2,100 genes had absolute LFC differences ≥1 (Fig. 6A, blue boxes). GO term analysis of the genes selected from the blue sections highlighted 173 biological processes that are regulated to a greater or lesser extent during secondary pneumococcal infection (Table S5). These GO processes were largely related to inflammatory, viral, host defense, and immune responses. We used Revigo (19) to summarize these biological processes and major categories represented by the circular heat plot in Fig. 6B. The four largest categories consisted of cytokine and inflammatory responses and epithelium-expressed genes inducing lymphocyte proliferation. A few categories seemed directly related to the HBEC microenvironment, involving intermediate filaments and collagen. We detail the specific genes involved in these four largest immune response categories, intermediate filaments, and collagen, by contrasting the LFC values of genes within these categories for pH1N1 infection (pH1N1 + HBEC vs HBEC) and secondary pneumococcal infection (pH1N1 + HBEC + EF3030 vs HBEC + EF3030) in Fig. S3. It appeared that the immune responses were largely influenced by specific interleukin and interferon genes relatively upregulated during secondary pneumococcal infection. However, intermediate filament changes were predominantly influenced by relatively upregulated keratin genes, and collagen metabolism by relatively upregulated matrix metalloproteinases.

### Influenza-induced secondary EF3030 infection in mouse lungs influences key genes triggered at the epithelial surface

**Fig 7:**
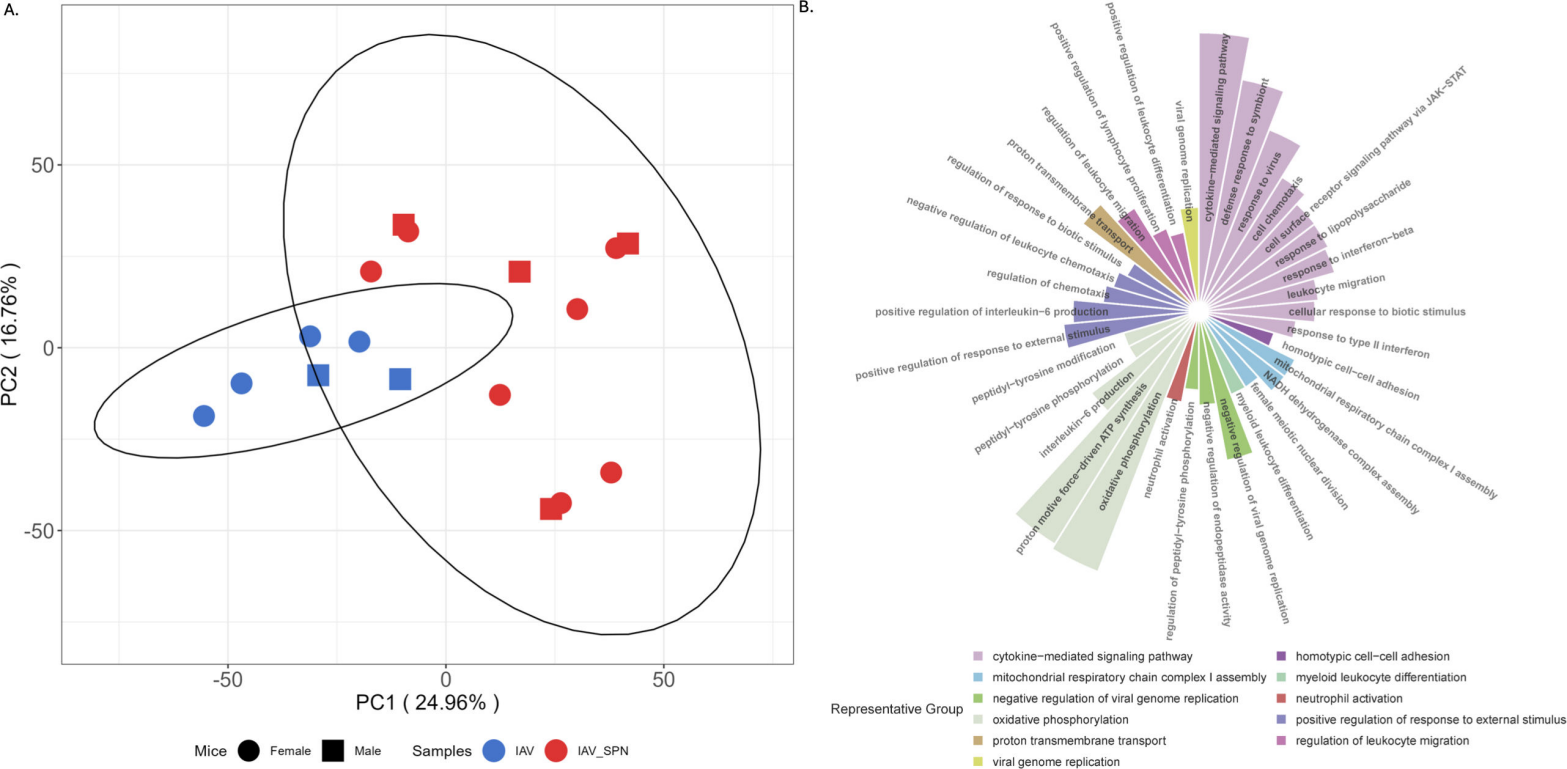
Overview of mouse lung RNA-seq transcriptomes. (**A**) PCA plot of infected mouse lung gene expression profiles. (**B**) Summarized REVIGO plot of GO biological processes enriched by DE genes from EF3030 + pH1N1-infected vs pH1N1-infected mouse lungs. Related categories have the same respective color. The size of individual highlights is proportional to GO enrichment-adjusted *P*-values.

To investigate the impact of secondary EF3030 infection from a whole lung perspective, we performed RNA-seq on pH1N1 and EF3030 + pH1N1-infected mouse lungs. The PCA of these samples showed partial overlap in their 95% confidence ellipses, suggesting the whole lung transcriptome was marginally affected by secondary EF3030 infection, similar to HBEC infections (Fig. 7A). Interestingly, infection timepoints of 12 h, 24 h, and 48 h showed no clear separation, suggesting that infection responses were largely consistent during the 12–48 h; hence, DE genes were estimated from an EF3030 + pH1N1 coinfection vs. pH1N1 monoinfection perspective, representing influenza-induced secondary pneumococcal infection. This revealed ~800 DE genes between EF3030 + pH1N1 vs pH1N1-infected lungs, consisting of many cytokines, interferon-related genes, immunoglobulin genes, serpins, miRNAs, and zinc-finger proteins. These ~800 genes enriched 43 GO biological processes (Table S6). A Revigo analysis of these identified immune cell pathways and signaling, such as cytokine activation, leukocyte and neutrophil infiltration, and negative regulation of viral genome replication (Fig. 7B). These categories reflect the influence of native mouse immune cells on pH1N1 during coinfection. Interestingly, there was also an impact on mitochondrial respiration and oxidative phosphorylation. Despite the dissimilarity between human HBECs and murine whole lungs, when comparing these ~800 DE genes with their homologs among the ~2,100 HBEC genes involved in influenza-induced secondary EF3030 infection, we found 30 genes that overlapped (Table S6). These included six interferon genes, chemokine ligands CCL3, CCL3L1, CCL5, CXCL9, and IL22RA2, receptors CD80 and TLR7, and matrix metalloproteinase MMP8. We observed the elevation of four of these interferon genes (IFNA2, IFNA6, IFNA7, and IFNA13) in lymphocyte proliferation (Fig. S3B), CCL5 in cytokine signaling (Fig. S3A and C), and even MMP8 in collagen metabolism (Fig. S3F). Similarly, it has been shown that MMP9 inhibition is protective of IAV infection in mice (20). Our findings suggest that the host epithelium triggers immunological processes and extracellular matrix degradation with specific genes, and these effects are maintained during further migration into whole lungs despite the presence of lymphocytes and neutrophils.

## DISCUSSION

Current gaps in knowledge exist regarding the adaptation of Spn to mucosal surface environments. We utilized two models of Spn infection on differentiated human pulmonary airway epithelium, and in murine lungs. Previous findings have also shown that Spn growth is increased by IAV infection of HBECs (9). The large increase in pneumococcal burdens during coinfection would require significant resources for pneumococcal growth and replication. We saw that the EF3030 transcriptome reflects this necessity through the regulation of a diverse panel of genes, mainly metabolic processes. Our findings showed that upon encountering the human lung epithelium, multiple scavenging and nutrient processing genes are upregulated to grow. However, during pH1N1 coinfection, several of these are no longer as important, likely due to the destructive effect of pH1N1 on the human lung epithelium and the release of previously sequestered nutrients.

We highlighted specific pneumococcal pathways showing responses to one of the three conditions we studied, namely, EF3030 in media only, HBEC + EF3030, and pH1N1 + HBEC + EF3030 (Fig. 3). We observed regulation specific to EF3030 encountering the HBECs in the form of zinc homeostasis and ascorbate metabolism. It was established that Spn requires zinc to survive and responds to environmental Zn^2+^ levels (21). This study also established in strain D39 that there are two Zn^2+^ uptake mechanisms, one involving the *acdA* gene, the other utilizing histidine triad proteins (Phts), and their functions are complementary (21, 22). Based on our expression data, there is a slight difference between EF3030 infection and pH1N1 coinfection among the Phts. This is interesting as it was shown that Phts require surface attachment to function and provide zinc (21), supporting our results that EF3030 is in a biofilm state during infection but not pH1N1 coinfection, and that potentially different mechanisms of zinc acquisition are involved during pH1N1 coinfection. Additionally, we had identified and experimentally verified that the ascorbate metabolism operon was a pneumococcal colonization signal in our prior *in vivo* mouse nasopharynx infection with strain TIGR4 (23). These together support the hypothesis that EF3030 infection of HBECs results in a biofilm-based surface colonization, until the introduction of pH1N1 potentially turning EF3030 invasive.

Multiple Spn regulons showed stark expression profiles specific to pH1N1 coinfection, namely fatty acid biosynthesis, amino acid metabolism, and pyrimidine metabolism (Fig. 3). The RegPrecise database asserts that pyrimidine metabolism in Spn TIGR4 is regulated (wholly or partially) through non-coding RNA mechanisms. It has been determined that deactivation of fatty acid biosynthesis results in a colony phase variation (24) and can also affect quorum sensing (25). It is quite possible that this allows EF3030 to leave its colonization state during pH1N1 coinfection and turn invasive. Our group and others have also previously shown that fatty acid biosynthesis is downregulated not only in the blood (23, 26, 27) but also in human plasma (27), further supporting the invasive switch. The pyrimidine metabolism regulon is fairly understudied in pneumococci apart from the mechanism of regulation (28). Downregulation of pyrimidine genes during coinfection could be due to the pH1N1-induced HBEC lysis, providing an excess of free nucleotides for uptake.

One of the most interesting and striking patterns of gene expression we observed was for NanR sialic acid utilization (Fig. 3), in the context of HBEC infection. A pattern repeated in lactose/galactose utilization. Utilization of these carbon sources is known to be favorable for biofilm formation (29), again suggesting an EF3030 biofilm status and a trend toward being in a colonization mode on HBEC without external influences such as immune cells or coinfection. We demonstrated that EF3030 ∆*nanR* resulted in the upregulation of other metabolic regulons, specifically in the absence of IAV. This validates the gene expression data and supports the hypotheses that excess influenza neuraminidase releases sialic acid into the lumen (30), thereby reducing the need for EF3030 NanR, an established pneumococcal regulon (31). However, studies that have shown the impact of this regulon during pneumococcal colonization and influenza coinfection implicate the regulator CiaR (EF3030_RS03775), neuraminidases NanA and NanB (EF3030_RS07895, EF3030_RS07950), and sialic acid transporter operon SatABC (EF3030_RS07920-EF3030_RS07930) (11, 14, 32). We verified that these genes are critical in our HBEC coinfection model, with CiaR being significantly upregulated only during pH1N1 coinfection and not monoinfection. Interestingly, the *satABC* genes, sometimes also referred to as *nanW*, *nanV,* and *nanU*, that are part of the whole NanR regulon, were significantly upregulated in both mono/coinfection (Fig. 3 NanR). However, we show that additional genes *nanPE* (EF3030_RS07935-EF3030_RS07940) are also involved, expanding the overall mechanism. Downregulation of the LacR regulon was also observed in D39 at high glucose concentrations (33). This potentially prepares EF3030 for transitioning into the glucose-rich bloodstream during pH1N1 coinfection. Moreover, the overall fitness improvement seen in EF3030 ∆*nanR* and its downregulation during coinfection suggests that the loss of these genes potentially permits EF3030 growth and invasiveness through a disinhibition of other EF3030 genes we identified (Fig. 4). However, this phenotype could only be observed in media or on HBEC only, and not in the murine lung (Fig. S2E); therefore, it is potentially noteworthy from a prophylactic perspective, but not after establishment of invasive pneumococcal disease. Preliminary influenza-induced cell damage permits improved pneumococcal metabolic activity and growth, but this is localized to the epithelium.

Another study by our group also showed that in such a pneumococcal state, the host response in a mouse nasopharynx model is also minimal (23). This is seen here in EF3030 on HBECs in both the EF3030 and host transcriptome. However, upon pH1N1 coinfection, EF3030 turns invasive and alters its metabolism to better suit the new environment. To better understand this, we correlated differentially expressed core pneumococcal genes from pneumococcus (TIGR4, D39, and 6A10)-infected murine lungs (invasive) relative to the nasopharynx (colonization), with DE genes of pH1N1 + HBEC + EF3030 (invasive) vs HBEC + EF3030 (colonization). We found 52/228 EF3030 DE genes that were shared in all strains and DE in the same direction and similar magnitudes (log_2_FC correlation *R* = 0.74). These included consistent downregulation of many of the same genes and operons from the NanR, LacR, and fructose/mannose metabolism regulons (Fig. 3), as well as upregulation of 2 distinct LysM peptidoglycan-binding domain proteins. In addition, all these carbon sources are acquired from epithelial host glycoconjugates (29). During pH1N1 coinfection, these are presumably no longer used as nutritional sources, allowing EF3030 to turn invasive. Such strong high expression and tight regulation of these genes could also contribute to EF3030 being a preferentially lung-associated pneumococcal strain. The presence of pH1N1 causes Spn proliferation with excess nutrients and even dispersion from biofilms into invasive states (34, 35), potentially also occurring for other pneumococcal strains during coinfection.

Some pneumococcal virulence factors in influenza superinfections were explored for essentiality in an *in vivo* CRISPRi-seq model, which showed that the pneumococcal capsule, together with *purA*, *bacA*, and *pacL* genes, was required for transmission, but interestingly not pneumolysin (7). We see in our data set that in EF3030, most of the genes in the capsular locus (EF3030_RS01695–EF3030_RS01745) were not significantly DE when they encountered the host lung epithelium, but their expression is elevated. We also saw that pneumolysin (EF3030_RS09370) was significantly downregulated when encountering the host lung epithelium, but *bacA* (EF3030_RS02235) had no changes in expression across all conditions. All the genes found relevant during influenza superinfection in CRISPRi-seq, except for *bacA*, a bacitracin susceptibility gene (likely due to it being identified *in vivo* in mice, which could potentially contain other lung microbes), follow suit with our data set in terms of expression trends. This, combined with known dispersion from biofilm and increased bacterial loads observed in our study and others, supports the possibility that colonizing EF3030 switches toward a dissemination phenotype during influenza coinfection.

Platt et al. have demonstrated using transcriptomics and proteomics that pneumococcal metabolism can be altered by IAV alone, not requiring the presence of the host (36). Certain aspects of Spn metabolism can be explained by direct interaction with IAV. For example, using proteomics, they identified a strong downregulation in pyrimidine metabolism in the presence of IAV, regardless of the host. We observed this same response in our EF3030 transcriptomics. However, while they saw galactose metabolism upregulated through LacR, we see it is transcriptionally downregulated. This may be due to interaction with the HBECs or the coinfection-induced environment.

Several host pathways identified by GO analyses mediate upregulation of diverse immune regulatory systems (Table S5) to counteract infection. These observations are consistent with other studies showing the involvement of dendritic cell-induced cytokine signaling and IL-17 signaling (15, 37). These data suggest that pH1N1 infection and EF3030 + pH1N1 coinfection result in the upregulation of cytokines and signaling to recruit host immune cells. On the other end of the spectrum, we observed that several downregulated genes (>2,500 genes) are related to ciliary beating and microtubule-based cell movement in our GO analysis, suggesting that this is a significant biological process in response to influenza infection, with and without EF3030. This phenotype was also observed in our experiments, which followed identical protocols of infection, where a marked decrease in cilia was observed with immunofluorescence (Fig. 5). Such ciliary regulation is consistent with prior influenza and pneumococcal infection studies (38), also involving upregulation of IL-17 and IFN genes seen in our data.

Apart from these broader immune processes, our deconvolution of shared host DE genes by their magnitudes revealed two processes unique to our experimental design, both of which related to relevant intercellular (keratin upregulation, Fig. S3E) and extracellular (matrix metalloproteinase upregulation, Fig. S3F) infection mechanisms. The coinfection exacerbations of these specific, largely understudied keratins are likely induced as a result of epithelial stress and damage (39) and could potentially cause epithelial layer remodeling leading to pulmonary fibrosis (39–41). The disruption of the epithelium is also associated with degradation of the extracellular matrix by elevated matrix metalloproteinase genes, potentially clearing the way for fibroblast infiltration. Alternatively, this transitional effect on barrier disruption also paves the way for invasive pneumococcal disease. Interestingly, when observed from a whole lung perspective in mice, the largest upregulated matrix metalloproteinase from the epithelium, MMP8, is still found upregulated across the coinfected lung. This suggests that as influenza-induced invasive pneumococcal disease progresses, a potential feedback loop may be triggered, whereby disruption of the epithelium/ECM allows infection of subepithelial myofibroblasts and endothelial cells, which, in turn, release more MMP8 and trigger further infection, now only being restrained by macrophages (42).

Although we identified various pneumococcal and host biological processes related to pH1N1 + Spn mono-/coinfection, we acknowledge certain limitations of our study. The use of only the primary HBEC system devoid of vasculature, immune cells, and surrounding tissue does not fully represent *in vivo* coinfection conditions. Complete gene expression profiling with RNA-seq was performed on only a single biological donor, although specific genes were confirmed in two other donors. Moreover, investigating the EF3030 transcriptome was not feasible in mouse lungs due to overwhelming mouse transcripts, as well as mouse lung-level responses that overshadowed epithelial-specific effects seen in HBECs. Despite these, the study identifies EF3030 gene expression in human lung epithelial cells in the context of colonization and influenza coinfection, capturing distinct metabolic adaptations in each. Our study and others have established the relevance of sialic acid regulation in the context of EF3030 monoinfection and influenza coinfection directly on the human epithelium but also in mouse models, and we expanded the pneumococcal influence on HBEC to consist of not just pneumococcal NanA and NanB but a multi-operon regulon whose composition varies among pneumococcal strains. We determined that NanR deletion enhances EF3030 growth during HBEC mono- and influenza coinfection, but not in murine lungs, suggesting a proliferative effect only in human epithelial environments.

From an epithelium perspective, influenza infection distinctly remodels the HBEC transcriptome, reducing ciliary function and increasing immune signaling. Coinfection also impacts mitochondrial respiration, oxidative phosphorylation pathways, and importantly, we observed an impact on epithelial keratin and matrix-metalloprotease gene families, potentially influencing extracellular matrix remodeling. In conclusion, the data presented here provide valuable insights into Spn EF3030 and pH1N1 mono-/coinfections and their mutual influence in host-pathogen interactions.

## MATERIALS AND METHODS

A detailed description of all experimental procedures, including reagent sources, ethics, and protocols, is provided in the supplemental materials. The methods are summarized below for brevity and build upon protocols established in our previous works.

### *In vivo* mouse infection and collection of lung samples

Mice were intranasally infected with IAV PR8 (H1N1 A/Puerto Rico/8/1934). Between 7 and 9 days post-infection (dpi), mice were challenged intratracheally with 10^7^ CFU of *Spn* strain EF3030 (serotype 19F). Infected mice were euthanized post*-Spn* infection at 12, 24, and 48 h. Lungs were cut into small pieces and placed into 5 mL of RNAprotect Bacteria Reagent and stored at −20°C for RNA isolation.

### Isolation, culture, and infection of differentiated human bronchial epithelial cells from human donors for RNA-seq and Nanostring nCounter platform validation

HBECs were isolated from donated human lung tissue, and cells were grown as previously described (9). Mature HBECs were washed, basally fed with PneumaCult-ALI basal medium, and infected with 100,000 plaque-forming units of A/California/07/2009 (pH1N1). After 72 h of H1N1 infection, HBECs were infected with 1,000 CFUs of Spn in 20 μL of physiological saline. For pneumococcal EF3030 control samples (no host cells), ~50,000 CFUs of EF3030 were cultured in ALI media for 6 h. After 6 hours of Spn infection, 1 mL of RNAprotect was added to all transwells, and then the whole transwells were stored in 5 mL of RNAprotect Bacteria Reagent in 50 mL Falcon tubes at −80°C for later extraction.

### RNA isolation, library construction, and sequencing

Transwell and mouse tissue samples were processed as previously described (23). RNA was then captured on the RNeasy Mini Kit columns with DNase treatment on column (Qiagen protocol). Ribosomal RNA was depleted, and 300 bp-insert RNA-seq Illumina libraries were constructed using ~1.0 μg of enriched mRNA that was fragmented and then used for the synthesis of strand-specific cDNA. RNA-seq was conducted on 150 nt paired-end runs of the Illumina NovaSeq 6000 platform using two or three biological replicates for each condition (23).

### RNA-seq data analyses

FASTQ files were mapped to their respective genomes, and normalized gene expression counts (VSTs) were estimated for PCA and rarefaction curves as previously described (23). DE genes were filtered using an FDR cutoff of ≤0.05 and an absolute LFC cutoff of ≥1. Common and unique DE genes for both species were determined using Upset plots (R package UpsetR [43]) and individual heatmaps of DE genes were generated based on Z-scores of VST counts (R package DESeq2 [17]).

### Orthology

Orthologous genes between EF3030 and TIGR4 were determined using PanOCT v3.23 (44) with the parameters -S Y -M Y -H Y -F 1.33 -c 0,25,50,75,100 -T.

### Bacterial gene regulon analysis

Bacterial DE gene lists were used to determine DE regulons using the online RegPrecise (45) and KEGG (46) databases for Spn TIGR4.

### GO analysis

GO ontologies and associated figures were estimated for all human DE genes from all comparisons (FDR ≤ 0.05 and an absolute LFC of ≥1) using ClusterProfiler version 4.0 (18).

### Knock-out mutants

Spn EF3030 mutants were generated via allelic exchange for each target operon (RS07920–RS07940, RS07890–RS07895), generating in-frame clean deletion mutants as previously described (23). The double mutant (EF3030: ΔRS07920–RS07940, ΔRS07890–RS07895) was generated using the single knock-out mutants as recipients for a second round of mutagenesis, following the same procedure. Primers for constructing the mutants are listed in Table S1.

### Pneumococcal NanoString nCounter panel and analysis

A custom set of 76 100-bp probes representing 72 pneumococcal genes (66 genes of interest + 6 housekeeping gene controls) was developed in collaboration with NanoString for targeted pneumococcal gene expression analysis. Data were processed using the NanoString nSolver software v4.0, comparing EF3030 mutants to WT, and pH1N1 + EF3030 infections to EF3030 infections for calculation of expression ratios.

## Data Availability

The data reported in this study are deposited in the Gene Expression Omnibus (GEO) database, https://www.ncbi.nlm.nih.gov/geo (accession numbers GSE225108, GSE294560, GSE294468, and GSE294726).

## SUPPLEMENTAL MATERIAL

Supplement Material msphere.00815-25-s0001.pdf Supplemental methods, figures, and table legends.

Table S1 msphere.00815-25-s0002.xlsx Mapping statistics for all samples.

Table S2 msphere.00815-25-s0003.xlsb Normalized (VST) gene expression counts.

Table S3 msphere.00815-25-s0004.xlsx Differential expression gene lists for EF3030.

Table S4 msphere.00815-25-s0005.xlsx Description of the custom EF3030 NanoString probe set.

Table S5 msphere.00815-25-s0006.xlsx Differential expression gene lists for HBEC.

Table S6 msphere.00815-25-s0007.xlsx Differential expression gene lists for mouse lungs.

ASM does not own the copyrights to Supplemental Material that may be linked to, or accessed through, an article. The authors have granted ASM a non-exclusive, world-wide license to publish the Supplemental Material files. Please contact the corresponding author directly for reuse.
