# Supplementary material for: Multispecies transcriptomics reveals influenza A virus modulation of Streptococcus pneumoniae EF3030 infection in human lung epithelium and murine lung
Source: mSphere. 2026 Jan 26;11(2):e00815-25. doi: 10.1128/msphere.00815-25 (PMC12931268; doi:10.1128/msphere.00815-25)
Supplement: Supplement Material — Supplemental methods, figures, and table legends. [file msphere.00815-25-s0001.pdf]

Supplemental Materials

Methods

Animal use. All experimental procedures involving mice were performed under the Institutional Animal Care and Use Committee (IACUC) approved protocol #22157 at The University of Alabama at Birmingham. Animal care and experimental protocols adhered to Public 578 Law 89-544 (Animal Welfare Act) and its amendments, Public Health Services 579 guidelines, and the Guide for the Care and Use of Laboratory Animals (U.S. Department 580 of Health & Human Services).

In vivo mouse infection and collection of lung samples. Male and female 9-week-old C57BL/6J mice were purchased from The Jackson Laboratory (Bar Harbor, ME). Mice were housed at 21°C in a 12-hour light/dark cycle and administered food and water ad libitum. Mice were anesthetized with 2.5% vaporized isoflurane in oxygen. Mice were intranasally infected by dropwise instillation into the nares with 250 plaque-forming units (PFU) of Influenza A virus PR8 (H1N1 A/Puerto Rico/8/1934) in 50 µL of phosphate buffered saline (PBS). Between 7-9 days post-infection (dpi), or after each animal had lost ~10% of their original body weight pre-infection, mice were anesthetized and challenged intratracheally with 10^7 CFU in 100 µL of PBS of *Spn* strain EF3030 (serotype 19F) by forced tracheal aspiration (1). Infected mice were monitored daily throughout, and euthanized post *Spn* infection at 12, 24, and 48 hours. For the collection of lung samples, lungs were first perfused with 3 mL of ice-cold PBS using a 27-gauge needle via the cardiac route (2) and then excised. The middle lung lobe was homogenized in 1 mL of PBS, serially diluted, and plated onto 5% sheep's blood agar plates for colony-forming unit (CFU) enumeration. The remaining tissue was cut into small pieces and

placed into 5 mL of RNAprotect® Bacteria Reagent (Qiagen#76506) and stored at -20 C° for RNA isolation. For the EF3030 ∆nanR vs. wt *in vivo* infections, ~9-week-old mice were infected with 250 plaque-forming units (PFU) of influenza A virus PR8, as above. Five days post-infection (dpi), or after each animal had lost ~20% of their original body weight pre-infection, mice were intratracheally instilled with ~10^7 CFU of Spn strain EF3030 (∆nanR or wt) in 100 ul of PBS by forced aspiration (1). All animals were monitored daily and sacrificed 24 hours post infection (hpi). Lungs were perfused with ~3 mL of ice-cold PBS using a 27-gauge needle via the cardiac route (2) and harvested. The middle lung lobe was homogenized in 1 mL of PBS, serially diluted, and plated onto 5% sheep's blood agar plates for colony-forming unit (CFU) enumeration.

Isolation, culture, and infection of differentiated human bronchial epithelial cells from human donors for RNA-seq and Nanostring nCounter platform validation. HBECs were isolated from donated healthy human lung tissue that was not viable for transplant provided by International Institute for the Advancement of Medicine (IIAM) and collected as previously described (3). Donors consisted of: YHA431 (RNA-seq) - a healthy adult male whose age was not available, AECI110 (nCounter) – a 33 year old healthy female, and AHJF214 (nCounter) – a 62 year old healthy female. Isolated cells were initially grown on 10 cm collagen coated dishes (Advanced Biomatrix) in BronchiaLife Complete Growth Media (BCGM LifeLine Cell Technologies) at 37°C with 5% CO_2. After reaching 80% confluence, cells were passaged to 6.5 mm permeable supports with a 4um pore size (Costar) coated with FNC coating mix (AthenaES) and maintained in the same media. Once cells reached 100% confluency, growth media was removed from the apical surface and the cells were differentiated with PneumaCult-ALI basal medium

(StemCell technologies). Basal media was replaced every day for seven days, and then three times weekly until the cell surface was 70% ciliated, usually occurring at approximately 18-21 days on air. During growth, cells were washed three times per week with PBS (Gibco) to remove excess mucus. All protocols using human tissue were reviewed by the UAB IRB as exempt as deidentified tissue procured through the IIAM. Mature HBECs were washed, basally fed with PneumaCult-ALI basal medium and infected with 100,000 plaque forming units of A/California/07/2009 (pH1N1). pH1N1 was obtained from the Center for Disease Control and Prevention. Spn was grown in beef heart infusion broth (Becton, Dickson and Company) supplemented with 10% catalase (Worthington) and 10% horse serum for 10-12 hours at 37°C with 5% CO_2 before quantification by OD600 reading and dilution in physiological saline. After 72 hours of H1N1 infection, HBECs were infected with 1,000 colony forming units of Spn in 20 uL of physiological saline. For pneumococcal EF3030 control samples (no host cells), ~50,000 CFUs of EF3030 were cultured in ALI media for 6 hours. After 6 hours of Spn infection, either 1ml of RNA protect was added to all transwells and then stored in 5ml of RNAprotect Bacteria Reagent in 50 ml falcon tubes at -80C or the apical surface was washed with 110uL of 10mM Tris HCl and 0.1mM EDTA. 10 uL of this wash was removed for quantification of Spn by growth on sheep's blood agar plates (Remel). 2.5 uL of 10% SDS and 2uL of 10% sodium deoxycholate was added to the remaining 100 uL of wash and incubated on the cell surface for 20 minutes at room temperature to ensure complete lysis of the HBECs and Spn. HBECs were scraped and collected in 600uL buffer RLT from the RNeasy mini kit (Qiagen) and RNA was extracted as below.

RNA isolation, library construction, and sequencing. Transwell and mouse tissue samples were thawed and the transwell membranes cut out using a scalpel. RNAprotect and isolated membranes or tissues were centrifuged at 10,000 rpm, to pellet the membrane and any dislodged cells, and the supernatant discarded. Pellets were then incubated in 100 µL of lysis buffer (10 µL of mutanolysin, 20 µL of proteinase K, 30 µL of lysozyme, 40 µL of TE buffer) for 10 minutes. Followed by mechanical disruption in 600 µL RLT buffer (RNeasy Mini Kit, Qiagen) containing 1% ß-mercaptoethanol, using a motorized pestle for 30 seconds (4). RNA was then captured on the RNeasy Mini Kit columns with DNase treatment on column (Qiagen protocol).

Extracted RNA was quantitated using a Bioanalyzer. Ribosomal RNA was depleted using the RiboZero rRNA Removal Kits for Gram-positive bacteria and/or for human/mouse/rat (Illumina). 300 bp-insert RNA-seq Illumina libraries were constructed using ~1.0 µg of enriched mRNA that was fragmented then used for synthesis of strand-specific cDNA using the NEBnext Ultra Directional RNA Library Prep Kit (NEB-E7420L). The cDNA was purified between enzymatic reactions and the size selection of the library performed with AMPure SpriSelect Beads (Beckman Coulter Genomics). The titer and size of the libraries was assessed on the LabChip GX (Perkin Elmer) and with the Library Quantification Kit (Kapa Biosciences). RNA-seq was conducted on 150 nt paired-end runs of the Illumina NovaSeq 6000 platform using two or three biological replicates for each condition (4).

RNA-seq data analyses. FASTQ files were mapped to their respective genomes using HISAT (5) for *Homo sapiens* or *Mus musculus* and Bowtie (6) for pneumococcal EF3030 and viral pH1N1 genomes. Gene expression counts for all samples were then estimated using HTseq (7). The counts tables were then used for analyses and estimation of

differentially expressed (DE) genes. Rarefaction curves were generated from counts data. Principal Component Analyses (PCAs) were generated in R based on normalized Variance Stabilized Transformation (VST) counts acquired using the DESeq2 R package (8). For human DE gene estimation, infected samples were compared to their respective uninfected control samples as the baseline using DEseq2 and filtered using an FDR cutoff of ≤0.05 and an absolute Log_2 Fold Change cutoff of ≥1. For mouse DE gene estimation, Spn and pH1N1 infected samples were compared to Spn infected samples with the same cutoffs. For bacterial DE gene estimation, each EF3030 infected sample was compared to EF3030 grown in ALI media as the baseline with the same cutoffs. Common and unique DE genes for both species were determined using Upset plots (R package UpsetR (9)) and individual heatmaps of DE genes were generated based on Z-scores of VST counts (R package DESeq2).

Orthology. Orthologous genes between EF3030 and TIGR4 were determined using PanOCT v3.23 (10) with the parameters -S Y -M Y -H Y -F 1.33 -c 0,25,50,75,100 -T.

Bacterial gene regulon analysis. Bacterial DE gene lists were used to determine DE regulons using the online RegPrecise (11) and KEGG (12) database for *Streptococcus pneumoniae* TIGR4.

Gene Ontology (GO) analysis. GO ontologies and associated figures were estimated for all Human DE genes from all comparisons (FDR ≤ 0.05 and an absolute Log_2 Fold Change of ≥1) using ClusterProfiler version 4.0 (13).

Knock-out mutants. *Streptococcus pneumoniae* EF3030 mutants were generated via allelic exchange. For each target operon (RS07920–RS07940, RS07890–RS07895),

upstream and downstream DNA fragments flanking the operon were amplified. These fragments were then fused at their 5′ and 3′ ends with the Janus cassette using a HiFi DNA Assembly Master Mix (NEB) to create mutagenic PCR constructs. EF3030 was transformed with the resulting constructs (100 ng/mL) in the presence of competence-stimulating peptide variant 1 (CSP-1). Kanamycin (300 mg/L) was used to select for Janus cassette integrants, while streptomycin (200 mg/L) selected for excision of the Janus cassette generating in-frame clean deletion mutants. The double mutant (EF3030: ΔRS07920–RS07940, ΔRS07890–RS07895) was generated using the single knock-out mutants as recipients for a second round of mutagenesis, following the same procedure. Primers for constructing the mutants are listed in Table S1. Growth curves were acquired by first streaking out the strains on blood agar plates (Remel) then incubating overnight at 37°C and 5.0% CO_2. Growth on these plates were then suspended in 5mL of Todd-Hewitt Broth (THY) Medium (Difco) and incubated at 37°C and 5.0% CO_2 until the suspension reached an OD600 of ~0.1. Then 20μL of this suspension was inoculated into 180μL of sterile THY medium within wells of 96-well plate (Falcon). 42 wells were used for each strain and 12 wells were left uninoculated. The plate was then incubated in a BioTek Cytation 5 Cell Imaging Multimode Reader for 14 hours at 37°C, 5.0% CO_2, and light shaking. OD620 of each well was measured every 15 minutes. The data were then exported, and growth rates were calculated for each well.

Pneumococcal NanoString nCounter panel and analysis. A custom set of 76 100bp probes representing 72 pneumococcal genes (66 genes of interest + 6 housekeeping gene controls) was developed in collaboration with NanoString for targeted pneumococcal gene expression analysis. The 66 genes of interest encompass diverse

carbon metabolic pathways, stress responses, surface proteins, and key virulence genes. Three pneumococcal genomes, TIGR4 (Accession:NC_003028), EF3030 (Accession:NZ_CP035897), and an in-house assembly and annotation of a cardiotropic ST700 strain (Accession:ERR3601523), were utilized to construct our probe set, and their conservation across the three genomes was assessed. Control genes were selected based on conserved pneumococcal housekeeping genes with the least variation in expression across prior RNA-seq data (4, 14, 15). 63 probes were identical in all three genomes, while 6 probes represented genes in two genomes and 7 probes were genome-specific. Among these, multiple probes were developed for highly variable but conserved genes, i.e. *pspA* (TIGR4:SP_0117, 3 probes), *nanA* (TIGR4:SP_1676, 2 probes), and *pspC* (TIGR4:SP_2190, 2 probes), to ensure detection across all three genomes. A list of all the probes used, their annotations, respective genomic coordinates, database sources, and gene metabolic and/or virulence status is described in Table S4. Data was processed using the NanoString nSolver software v4.0 comparing EF3030 mutants to wildtype, and pH1N1+EF3030 infections to EF3030 infections for calculation of expression ratios. Normalization included a negative control count thresholding value of 30, positive control normalization using geometric mean with default settings, excluding housekeeping genes that were found to vary in expression in our assays.

Supplemental Figures and Legends

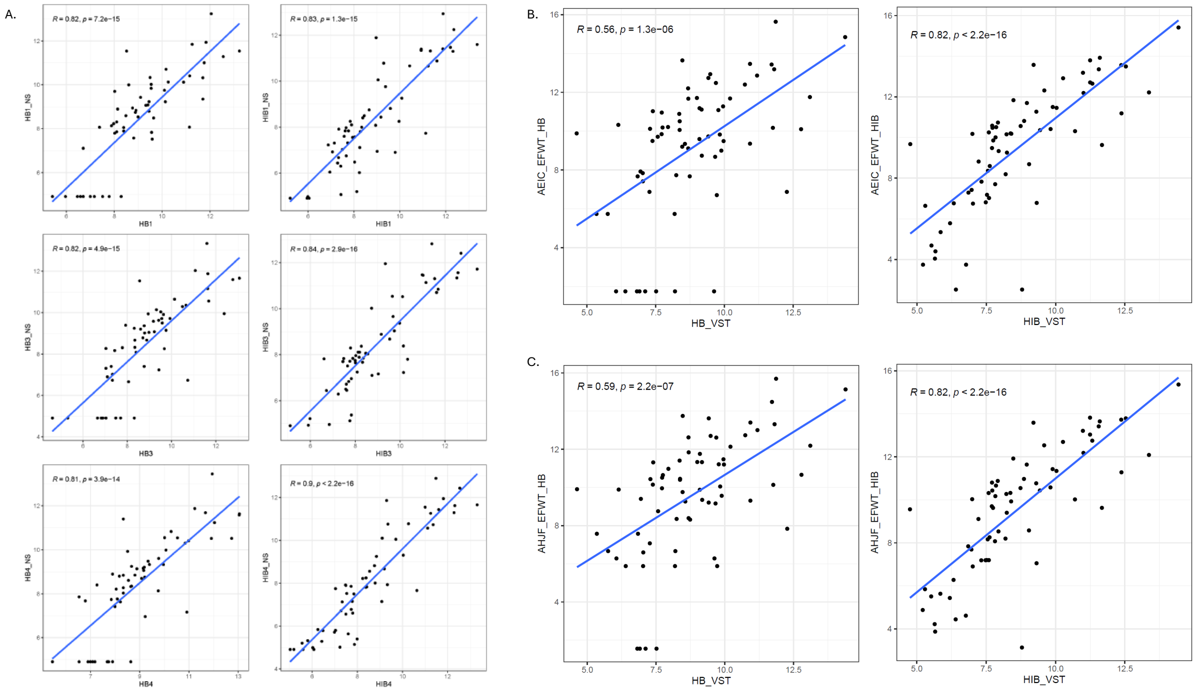

Figure S1. Spearman correlations of normalized expression values of specific EF3030 genes from RNA-seq and Nanostring nCounter. X axes are DESeq2 Variance Stabilized Transformation (VST) counts. Y axes are log_2 normalized nCounter expression values. A) RNA-seq vs nCounter correlations of specific EF3030 genes of the same sequenced transwell samples from Figure 1A. B) Correlations of average RNA-seq expression values from sequenced transwell samples from Figure 1A vs average nCounter expression values from a biological replicate of HBEC donor AEIC. C) Correlations of average RNA-seq expression values from sequenced transwell samples from Figure 1A vs average nCounter expression values from a biological replicate of HBEC donor AHJF. Average values were used as RNA-seq was not performed on donors AEIC and AHJF.

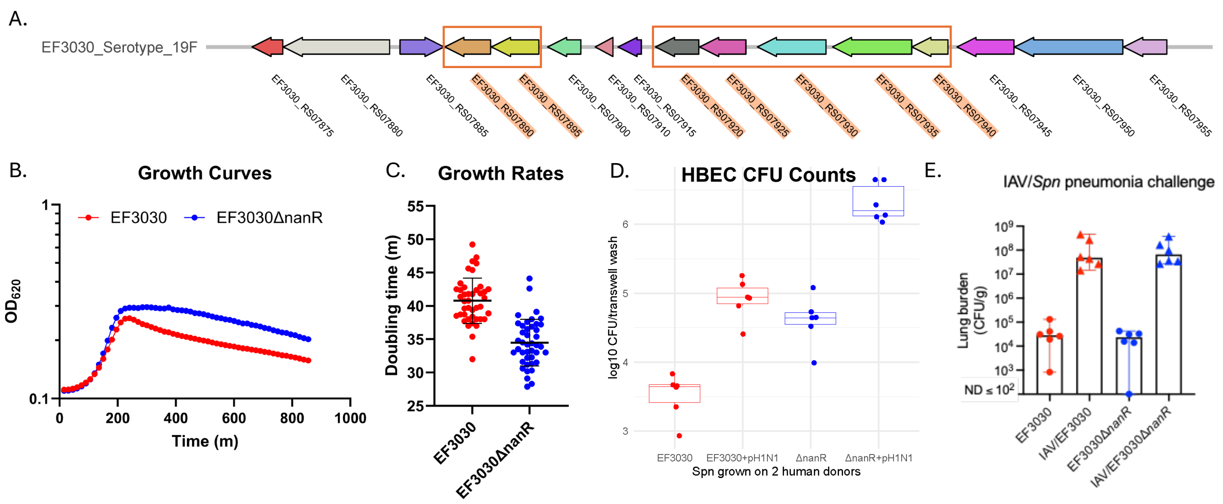

Figure S2. EF3030 wildtype vs. EF3030 Δ*nanR* growth kinetics. A) Overview of the EF3030 NanR regulon locus with knocked-out genes and operons highlighted in orange. B) Growth curves of EF3030 w.t. vs. EF3030 Δ*nanR* showing improved fitness upon NanR deletion in media. B) Doubling times of EF3030 w.t. vs. EF3030 Δ*nanR* in media. C) Transwell colony forming unit (CFU) counts for EF3030 w.t. and EF3030 Δ*nanR* grown on HBEC from two human donors, with and without pH1N1. E) CFU counts of *in vivo* murine lung infections of EF3030 w.t. vs. EF3030 Δ*nanR* with/without prior pH1N1 infection.

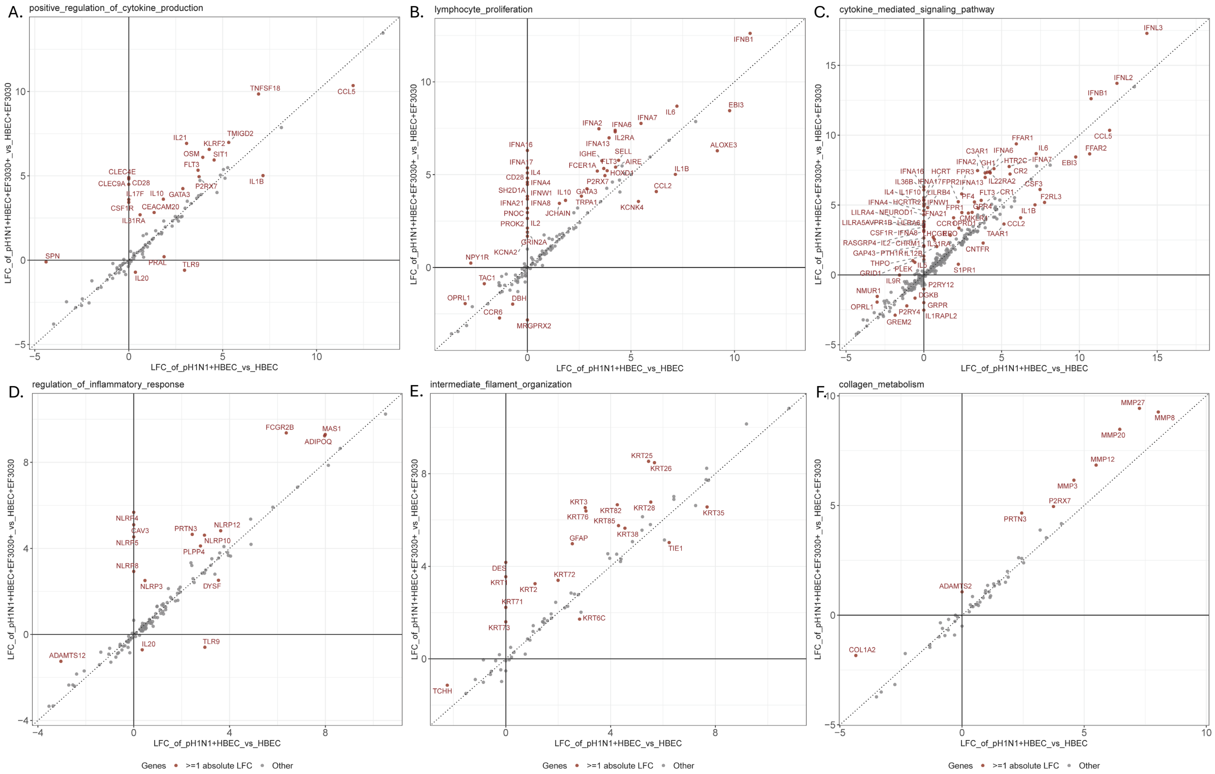

Figure S3. RNA-seq Log$_2$ Fold Change (LFC) scatterplots of genes involved in six GO biological processes enriched during influenza-induced HBEC secondary EF3030 infection. A-D) Plots of the 4 largest immunology GO processes from Figure 6B. E-F) Plots of the 2 epithelium-related GO processes from Figure 6B. Genes labeled in red had absolute LFC changes ≥1 from blue boxes in Figure 6A.

Supplemental Tables

Table S1. Mapping statistics for all samples. Mutant primers used in EF3030 Δ*nanR* construction.

Table S2. Normalized (VST) gene expression counts for all samples for each species of Spn EF3030, Human and Mouse.

Table S3. Differential expression (DE) gene lists (sheets have the prefix DE), their associated enriched biological pathways for EF3030 (sheets have the prefix Path), and a complete list of pathways.

Table S4. Description of the custom EF3030 NanoString probe set, and DE values for EF3030 Δ*nanR* vs EF3030 w.t. comparisons.

Table S5. DE gene lists (sheets have the prefix DE), their associated enriched GO biological processes for HBECs (sheets have the prefix GO), and a sheet of 173 GO pathways for the ~2100 genes of Figure 6A (labeled GO-SecondaryPneumoInf).

Table S6. DE gene list (sheet has the prefix DE), their associated enriched GO biological processes for mouse lungs (sheet has the prefix GO), and a list of mouse to human homologs of the DE genes.

References

1. Nielsen TB, Yan J, Luna B, Spellberg B. Murine Oropharyngeal Aspiration Model of Ventilator-associated and Hospital-acquired Bacterial Pneumonia. J Vis Exp. 2018(136).

2. Davenport ML, Sherrill TP, Blackwell TS, Edmonds MD. Perfusion and Inflation of the Mouse Lung for Tumor Histology. J Vis Exp. 2020(162).

3. Fulcher ML, Gabriel S, Burns KA, Yankaskas JR, Randell SH. Well-differentiated human airway epithelial cell cultures. Methods Mol Med. 2005;107:183-206.

4. D'Mello A, Riegler AN, Martinez E, Beno SM, Ricketts TD, Foxman EF, et al. An in vivo atlas of host-pathogen transcriptomes during Streptococcus pneumoniae colonization and disease. Proc Natl Acad Sci U S A. 2020;117(52):33507-18.

5. Kim D, Langmead B, Salzberg SL. HISAT: a fast spliced aligner with low memory requirements. Nat Methods. 2015;12(4):357-60.

6. Langmead B, Trapnell C, Pop M, Salzberg SL. Ultrafast and memory-efficient alignment of short DNA sequences to the human genome. Genome Biol. 2009;10(3):R25.

7. Anders S, Pyl PT, Huber W. HTSeq--a Python framework to work with high-throughput sequencing data. Bioinformatics. 2015;31(2):166-9.

8. Love MI, Huber W, Anders S. Moderated estimation of fold change and dispersion for RNA-seq data with DESeq2. Genome Biol. 2014;15(12):550.

9. Conway JR, Lex A, Gehlenborg N. UpSetR: an R package for the visualization of intersecting sets and their properties. Bioinformatics. 2017;33(18):2938-40.

10. Fouts DE, Brinkac L, Beck E, Inman J, Sutton G. PanOCT: automated clustering of orthologs using conserved gene neighborhood for pan-genomic analysis of bacterial strains and closely related species. Nucleic Acids Res. 2012;40(22):e172.

11. Novichkov PS, Kazakov AE, Ravcheev DA, Leyn SA, Kovaleva GY, Sutormin RA, et al. RegPrecise 3.0--a resource for genome-scale exploration of transcriptional regulation in bacteria. BMC Genomics. 2013;14:745.

12. Kanehisa M, Furumichi M, Sato Y, Matsuura Y, Ishiguro-Watanabe M. KEGG: biological systems database as a model of the real world. Nucleic Acids Res. 2025;53(D1):D672-D7.

13. Wu T, Hu E, Xu S, Chen M, Guo P, Dai Z, et al. clusterProfiler 4.0: A universal enrichment tool for interpreting omics data. Innovation (Camb). 2021;2(3):100141.

14. Im H, Kruckow KL, D'Mello A, Ganaie F, Martinez E, Luck JN, et al. Anatomical Site-Specific Carbohydrate Availability Impacts Streptococcus pneumoniae Virulence and Fitness during Colonization and Disease. Infect Immun. 2022;90(1):e0045121.

15. Shenoy AT, Brissac T, Gilley RP, Kumar N, Wang Y, Gonzalez-Juarbe N, et al. Streptococcus pneumoniae in the heart subvert the host response through biofilm-mediated resident macrophage killing. PLoS Pathog. 2017;13(8):e1006582.
